# The relationship between cardiovascular health and common mental disorders among adults aged ≤ 50 years: a systematic review of observational studies

**DOI:** 10.1186/s12888-026-08281-w

**Published:** 2026-06-16

**Authors:** Audrey Moyo, Theresa Munyombwe, Nasheeta Peer, Siyabonga Eric Dubazana, Nokwanda Sithole, Innocent Maposa, Darshini Govindasamy

**Affiliations:** 1 Division of Epidemiology and Biostatistics, Department of Global Health, Faculty of Medicine and Health Sciences, Stellenbosch University, Cape Town, South Africa; 2 Leeds Institute of Cardiovascular and Metabolic Medicine, University of Leeds, Leeds, UK; 3 Leeds Institute for Data Analytics, University of Leeds, Leeds, UK; 4 Non-Communicable Diseases Research Unit, South African Medical Research Council, Durban, South Africa; 5 Department of Medicine, Faculty of Health Sciences, University of Cape Town, Cape Town, South Africa; 6 Health Systems Research Unit, South African Medical Research Council, Cape Town, South Africa; 7 Health Economics and Epidemiology Research Office (HE2RO), Wits Health Consortium, Johannesburg, South Africa; 8 Faculty of Health Sciences, University of the Witwatersrand, Johannesburg, South Africa; 9 SAMRC/Wits Centre for Health Economics and Decision Science -PRICELESS SA, Division of Wits Health Consortium, WITS School of Public Health, Faculty of Health Sciences, University of the Witwatersrand, Johannesburg, South Africa; 10 Wits School of Public Health, Faculty of Health Sciences, University of the Witwatersrand, Johannesburg, South Africa

**Keywords:** Common mental disorders, Cardiovascular health, Depression, Anxiety, Systematic review, Observational studies, Mental health

## Abstract

**Background:**

Depression and anxiety are common among younger adults and contribute to adverse physical and social outcomes. Evidence suggests a potential bidirectional relationship between common mental disorders (CMDs) and cardiovascular health (CVH). However, this has not been directly established. This review examined the relationship between CVH and CMDs in individuals aged 15–50 years.

**Methods:**

MEDLINE (PubMed), APA PsycINFO, Web of Science, and CINAHL (EBSCO) were searched from inception to 4 November 2024. Observational studies reporting depressive or anxiety symptoms and CVH defined by Life’s Simple 7, Life’s Essential 8, or adapted composite indices were eligible. Study quality was appraised using the Newcastle-Ottawa Scale and a narrative synthesis was conducted.

**Findings:**

Of 25 full-text articles assessed, 11 studies (*n* = 731,650; mean age 22–48 years) met the inclusion criteria. Nine were conducted in the United States, one in China, and one in Puerto Rico. Seven cross-sectional studies reported an inverse association between CVH and CMDs, with higher CVH associated with lower odds or prevalence of depressive and anxiety symptoms. Four studies, including one cohort, found that CMDs were associated with lower CVH, reflected by higher odds of suboptimal CVH, lower prevalence of ideal CVH, and reduced CVH scores.

**Conclusions:**

Findings suggest a potential bidirectional relationship between CVH and CMDs among adults, though this is inferred from separate analyses rather than directly established using the same studies. Integrated strategies promoting physical activity, a healthy diet, and stress management, alongside primary care initiatives incorporating behavioural support and screening for cardiovascular and mental health risks, may benefit both conditions. Interpretation is limited by the predominance of cross-sectional studies and heterogeneity in study designs, with most studies conducted in high-income settings. Future research should prioritise longitudinal designs and apply diverse analytical methods to better clarify directionality.

**Registration:**

PROSPERO (CRD42024621494).

**Supplementary Information:**

The online version contains supplementary material available at 10.1186/s12888-026-08281-w.

## Introduction

Common mental disorders (CMDs), namely depression and anxiety, cause emotional distress that disrupts daily functioning [1]. They impose a long-term burden on adults aged ≤ 50 years, adversely affecting health, relationships, and economic wellbeing, and increasing the risk of morbidity and premature mortality [2]. In 2021, the global prevalence of depression among individuals aged 15–49 years ranged from 3.4% to 6.2% [3], while the prevalence of anxiety remained consistently high, ranging from 5.3% to 5.8% [3].

Given this burden of CMDs, it is crucial to understand their associations with other health conditions, including evidence of a potential bidirectional relationship with cardiovascular diseases (CVDs). One study reported an 8% and 26% higher relative risk of incident depression and anxiety, respectively, among individuals with CVDs, defined by self-reported diagnosis or medical claims [4]. Conversely, meta-analyses showed that adults with depression and anxiety were at increased risk of developing CVDs by 16% and 52%, respectively [5, 6].

Among adults aged ≤ 50 years, CVD incidence has remained stable or increased in recent decades, alongside rising cardiovascular risk factors [7]. Between 1990 and 2019, the age-standardised prevalence of CVDs among individuals aged 15–39 years increased from 1477.5 to 1645.3 per 100,000 population [8]. Although clinical CVD may be less common in individuals under 50 years of age [9], many exhibit risk factors such as hypertension, obesity, physical inactivity, and poor diet [7]. These exposures accelerate atherosclerosis, characterised by arterial plaque build-up, which underlies major conditions such as myocardial infarction and stroke [9, 10]. As atherosclerosis often begins in childhood and progresses silently with age, promoting cardiovascular health (CVH) early is critical to preventing premature morbidity and mortality [9].

The CVH–CMD relationship is complex, involving biological mechanisms (inflammation, oxidative stress, neurochemical changes, endothelial dysfunction, and platelet activation) and behavioural pathways (tobacco smoking, physical inactivity, and substance abuse) [11]. These mechanisms may increase cardiovascular risk and exacerbate symptoms of depression and anxiety [11]. To promote CVH and prevent CVDs, the American Heart Association (AHA) introduced the concept of CVH [12], defined by eight metrics known as *Life’s Essential 8 (LE8)*: blood pressure, blood lipids, body mass index (BMI), blood glucose, physical activity, tobacco smoking, diet, and sleep health [13]. LE8 updates the earlier *Life’s Simple 7 (LS7)* construct by adding sleep health. These metrics allow CVH and wellness to be monitored over time.

Low CVH is associated not only with increased CVD risk but also with adverse mental health outcomes. For instance, one study found that higher CVH was associated with lower odds of depressive and anxiety symptoms [14], while another focusing on adults ≤ 50 years reported lower CVH among those with CMDs [15]. A systematic review by Ogunmoroti et al. [16] suggested a bidirectional relationship between depressive symptoms and CVH. However, that review focused on adults, without examining the topic in relation to anxiety or discussing the methodological approaches used in such studies.

Addressing this gap, we extend the work by Ogunmoroti [16] by focusing on individuals aged ≤ 50 years, including both depression and anxiety, and examining the methodological approaches used in previous studies. Previous studies on CVH and CMDs often adopt wider age ranges than those defined for young adults. For example, Kwapong et al. [17] defined young adults as 18–49 years. We therefore included adults aged 15–50 years. Maintaining good CVH during this period supports healthier ageing [18] and is essential for sustaining healthcare systems by reducing future CVD burden and related costs, while also supporting economic productivity given the central role this group plays in the workforce [19]. Furthermore, understanding this relationship can inform strategies for primordial prevention and management of non-communicable diseases (NCDs), supporting the United Nations Sustainable Development Goal 3.4 to reduce premature NCD mortality [20]. Finally, given the complexity of this relationship, diverse methodological approaches may help reveal underlying epidemiological patterns. This review therefore aims to synthesise existing evidence on this relationship among adults ≤ 50 years and to critically evaluate the methodological approaches used, thereby informing future research, policy and practice.

## Methods

### Search strategy and selection criteria

This systematic review followed the Preferred Reporting Items for Systematic Reviews and Meta-Analyses (PRISMA) 2020 guidelines [21]. The protocol was registered on PROSPERO (CRD42024621494), and any amendments made after registration are documented in the PROSPERO record. Independent searches were performed across MEDLINE (PubMed), APA PsycINFO, Web of Science, and CINAHL (EBSCO) from database inception to 4 November 2024. Studies reporting on depressive or anxiety symptoms and CVH were eligible. The search combined Medical Subject Headings (MeSH) terms such as “mood disorders,” “anxiety,” and “heart disease risk factors,” with keywords including “CVH,” “composite cardiovascular risk factors,” “depression,” “anxiety,”. Age-related terms included “adolescents”, “young adults”, “adults”. Full search strategies are shown in Tables S1–S4. No language restrictions were applied. Searches were conducted by one independent reviewer (AM), with support from a librarian.

References were imported into EndNote for duplicate removal. Records were then transferred to Rayyan software for screening [22]. Title and abstract were screened independently by three reviewers (AM, SED, NS). Full-text screening was completed by one reviewer (AM), with oversight and quality checking by co-authors (DG, IM, TM). The PRISMA 2020 Shiny app was used to generate the screening flow diagram [23]. Studies were eligible if they met four criteria. Participants included adults aged 15–50 years. Broader adult populations were included if the mean age fell within this range and subgroup analyses were reported. Eligible studies used observational designs, including cross-sectional, cohort, or case-control designs. These studies examined the relationship between CVH and CMDs (depressive or anxiety symptoms) in either direction. Only studies published in peer-reviewed journals in English or with available translation were included.

CMDs had to be assessed using validated diagnostic criteria (e.g., Diagnostic and Statistical Manual of Mental Disorders), standardised screening tools (e.g., Patient Health Questionnaire-9 (PHQ-9) for depressive symptoms, Generalized Anxiety Disorder-7 (GAD-7) for anxiety symptoms), or self-reported diagnosis or symptoms [24, 25]. CVH had to be measured using either the AHA metrics [12, 13] or an adapted composite index based on cardiovascular risk factors [17].

Studies were excluded if participants were younger than 15 or older than 50 years, or if relevant subgroup analyses were not reported. Studies examining only individual cardiovascular risk factors or useing experimental designs, including randomised controlled trials, were also excluded. Theses, book chapters, conference abstracts, reviews, editorials, and commentaries were excluded. Formal reference list screening was not conducted. However, the search strategy was designed to be comprehensive and capture all studies from the most relevant prior review [16].

### Data analysis

The methodological quality of included studies was assessed using the Newcastle-Ottawa Scale (NOS) for cohort studies [26] and its modified version for cross-sectional studies [27] (Text S1). Quality appraisal for both designs was based on three broad criteria: selection of study groups, comparability of groups, and ascertainment of either the exposure or outcome [26, 27]. Cohort studies were scored out of nine stars, while cross-sectional studies were scored out of seven [26, 27]. Studies were categorised as poor (≤ 3 stars), moderate (4–6 stars), or high quality (≥ 7 stars) [26, 27]. Appraisals were conducted by one independent reviewer (AM), with oversight and quality checking by co-authors (DG, IM, TM).

Data extraction was performed by one reviewer (AM) using Microsoft Excel, with oversight and quality checking from co-authors (DG, IM, TM). A predesigned form, informed by the STROBE (Strengthening the Reporting of Observational Studies in Epidemiology) reporting criteria, was used to extract the following information: authors, study location, design, data source, participants’ characteristics, sample size, mean age, outcomes, exposures, covariates, statistical models, and results. Due to heterogeneity in definitions of outcomes, exposures, and measures of associations, a narrative synthesis approach was used to analyse the findings. This heterogeneity arose from differences in CVH measurement, including the use of LS7, LE8, and modified indices with varying scoring systems. It also reflected differences in CMD assessment tools, which differ in the number of items, scoring systems, and thresholds used to define symptoms. Differences in analytical approaches further limited the comparability of effect estimates across studies.

The narrative synthesis involved grouping studies by exposure and outcome direction, summarising methodological characteristics, and comparing the consistency and direction of reported associations. Forest plots were used to visually present the effect estimates reported in included studies, illustrating the direction and consistency of associations. Effect estimates were re-expressed by reversing group comparisons to ensure consistent direction of association across studies for interpretive purposes.

### Role of the funding source

The authors received no funding to conduct this study.

## Results

### Literature search

**Fig. 1:**
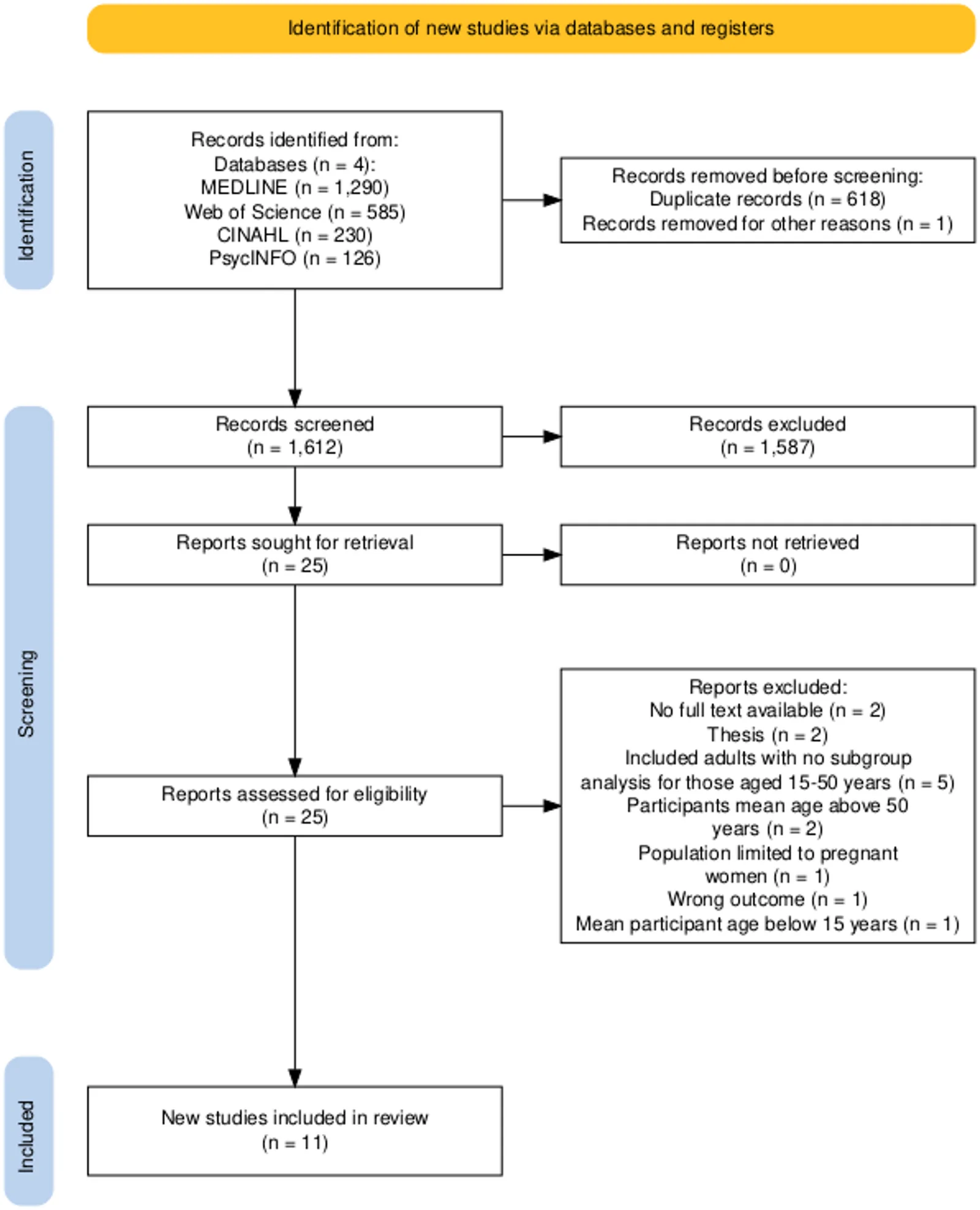
PRISMA flow diagram of study selection

The search across four databases yielded 2231 records. After removing duplicates and articles without English translations, 1612 studies remained for title and abstract screening. Of the 25 full-text articles assessed, 11 met the eligibility criteria. Full details of the search and screening process are presented in Fig. 1.

### Study characteristics

Table 1a summarises the characteristics of the 11 included studies, comprising 731,650 participants. Mean ages ranged from 22 to 48 years. Ten studies used a cross-sectional design [14, 15, 17, 28–34], and one was a prospective cohort study [35]. Among the cross-sectional studies, seven focused on participants aged ≥ 20 years [14, 28–30, 32–34], while three specifically targeted young adults aged 18–29 years [31], 18–34 years [15], and 18–49 years [17]. Although some studies included older adults, subgroup or sensitivity analyses stratified by age were conducted. This review focused on results from subgroups within the 15–50-year age range, including 20–44 years [28, 33], 20–39 years [29, 30, 34], 20–34 and 35–49 years [32], and 20–47 years [14]. The cohort study included participants aged 18–30 years at baseline, with a 20-year follow-up (ages 38–50 years) [35]. Geographically, nine studies were conducted in the United States [14, 15, 17, 28, 29, 32–35], one in China [30], and one in Puerto Rico [31].

**Table 1a:** Characteristics of included studies

Author	Study Location	Study Design	Data Source	Participants	Sample Size	Mean Age (years)
Bai and Guo, 2024 [28]	United States	Cross-sectional	NHANES 2005–2018	Aged ≥ 20 years; 48.83% (*n* = 10,386) aged 20–44 years; 48.51% (*n* = 10,601) female	22,149	45.76 (SE = 0.25)
Carroll et al., 2020 [35]	United States	Prospective cohort	CARDIA 1985–2006	Aged 18–30 years at baseline; aged 38–50 at year 20; 57% female	3086	45 (SD = 3.6)
Kwapong et al., 2023 [17]	United States	Cross-sectional	BRFSS 2017–2020	Aged 18–49 years; CVH assessed in 2017 & 2019; 49.7% female	593,616	34.7 ± 9.0
Li and Dai, 2024 [29]	United States	Cross-sectional	NHANES 2005–2018	Aged ≥ 20 years; LS7: 39.21% aged 20–39 years, 48.19% female; LE8: 34.62% aged 20–39 years, 51.53% female	*n* = 21,370 (LS7); *n* = 24,871 (LE8)	45.89 (SE = 0.28, LS7); 47.93 (SE = 0.26, LE8)
Li et al., 2015 [30]	China	Cross-sectional	Jidong community; Tangshan City (2013)	Aged ≥ 20 years; 3326 female	6851	42.1
Patterson et al., 2022 [15]	United states	Cross-sectional	Emory Healthy Aging Study 2015–2019	Aged 18–34 years; no CVD history; 82.7% female	875	28.3 (SD = 4.1)
Rosal et al., 2024 [31]	Puerto Rico	Cross-sectional	PR-OUTLOOK 2020–2023	Aged 18–29 years; 60.9% female	2156	22.6 (SD = 3.1)
Xu et al., 2024 [14]	United States	Cross-sectional	NHANES 2007–2012	Aged ≥ 20 years; not pregnant; LE8 categories: low (48.76%), moderate (48.46%), high (59.24%) female	13,028	47.56
Zeng et al., 2024 [32]	Unted States	Cross-sectional	NHANES 2007–2018	Aged ≥ 20 years; not pregnant; no cancer or CVD; 51.89% females	16,362	44.72 (0.27)
Zhang et al., 2019 [33]	United States	Cross-sectional	NHANES 2007–2014	Aged ≥ 20 years; not pregnant; BMI ≥ 18.5 kg/m^2^; no CVD & cancer; 50.4% female	14,561	44.6 (SE = 0.31)
Zhao et al., 2024 [34]	United States	Cross-sectional	NHANES 2011–2020	Aged ≥ 20 years; not pregnant; BMI ≥ 18.5 kg/m^2;^ no CVD history; 76.24% female	12,725	46.40 (SE = 0.37)

ALSPAC, Avon Longitudinal Study of Parents and Children; BMI, body mass index; BRFSS, Behavioral Risk Factor Surveillance System; CARDIA, Coronary Artery Risk Development in Young Adults; CVD; cardiovascular disease; CVH, cardiovascular health; kg/m^2^, kilograms per meter squared; LE8, Life’s Essential 8; LS7, Life’s Simple 7; NHANES, National Health and Nutrition Survey; PR-OUTLOOK, Puerto Rico Young Adults’ Stress, Contextual, Behavioral & Cardiometabolic Risk; SD, standard deviation; SE, standard error

**Table 1b:** Characteristics of included studies (cont’d)

Author	Outcome(s)	Exposure(s)	Covariates	Statistical models	Significant findings
Bai and Guo, 2024 [28]	Depressive symptoms (PHQ-9 ≥ 10)	CVH; LE8; continuous (0–100); categorical: low (0–49); moderate (50–79), ideal (80–100)	Age, gender, race, education, marital status, poverty-to-income ratio, CVDs (taking cardiovascular agents or self-reported diagnoses of heart failure, angina, congestive heart failure), chronic kidney disease, arthritis, energy intake, antidepressants, antipsychotics	Logistic regression	Among participants aged < 45 years, higher LE8 scores were associated with lower odds of depressive symptoms
Carroll et al., 2020 [35]	CVH; modified LS7 (0–12), excluding smoking	Depressive symptoms (CES-D-20 ≥ 16); trajectories (no, subthreshold, decreasing, increasing, high)	Age, sex, race, education	Latent class analysis (trajectories); GLMs	Increasing depressive symptoms trajectories over time were associated with low CVH in mid-adulthood.
Kwapong et al., 2023 [17]	CVH; modified LS7: optimal (< 2 cardiovascular risk factors), suboptimal (≥ 2 cardiovascular risk factors)	Depressive symptoms (self-report diagnosis history by a healthcare professional)	Age, sex, race, education, employment, health coverage, marital status, income	Logistic regression	Depressive symptoms were associated with higher odds of suboptimal CVH
Li and Dai, 2024 [29]	Depressive symptoms (PHQ-9 ≥ 10)	CVH; LS7 (low 0–7, moderate 8–10, ideal 11–14); LE8 (low 0–49, moderate 50–79, ideal 80–100)	Age, gender, race, education, marital status, income-to-poverty ratio, alcohol use, CVDs (coronary artery disease, congestive heart failure, myocardial infarction, stroke, angina pectoris), neoplasms, thyroid, liver diseases, arthritis, tumours	Logistic regression; RCS; ROC; DeLong tests	Among participants aged < 40 years, higher CVH (both LS7 and LE8) were associated with lower odds of depressive symptoms.
Li et al., 2015 [30]	Depressive symptoms (CES-D-20 ≥ 16)	CVH; LS7 (quartiles: ≤8, 9–10, 11, 12–14)	Age, gender, marital status, alcohol use, income, education, history of myocardial infarction, stroke, and cancer	Logistic regression	Among participants aged < 40 years, higher CVH scores were associated with lower odds of depressive symptoms
Patterson et al., 2022 [15]	CVH; LS7 (low 0–7, moderate 8–11, ideal (12–14)	Anxiety symptoms (GAD-7 ≥ 5); depressive symptoms (PHQ-9 ≥ 5)	Age, sex, race, ethnicity, income, education	Logistic regression; linear regression	Anxiety and depressive symptoms, individually or combined, were associated with low CVH
Rosal et al., 2024 [31]	CVH; LE8 (suboptimal: low 0–49 or moderate 50–79; ideal 80–100	Depressive symptoms (CES-D-10 ≥ 10); anxiety symptoms (STAI-10 ≥ 27)	Age, sex, marital status, education, health insurance, social status, psychological factors	Logistic regression; dominance analysis	Depressive or anxiety symptoms were associated with low CVH and higher odds of suboptimal CVH
Xu et al., 2024 [14]	Depressive symptoms (PHQ-9 score ≥ 10); anxiety symptoms (≥ 7 anxious days)	CVH; LE8 (low 0–49, moderate 50–79, ideal ≥ 80)	Age, sex, race, marital status, poverty, education	Logistic regression; RCS	Among participants < 47.6 years, each unit increase in LE8 was associated with 5% lower odds of depressive symptoms and 2% lower odds of anxiety symptoms
Zeng et al., 2024 [32]	Depressive symptoms (PHQ-9 ≥ 10)	CVH; LE8 (low 0–49, moderate 50–79, ideal 80–100)	Age, gender, race/ethnicity, education, marital status, poverty income ratio, and alcohol use	Logistic regression	Low and moderate CVH were associated with increased odds of depressive symptoms; strongest in participants aged 35–49 years
Zhang et al., 2019 [33]	Depressive symptoms (PHQ-9 ≥ 10)	CVH; LS7 (low 0–4, moderate 5–9, ideal 10–14)	Age, sex, race, ethnicity, education, alcohol use	Multinomial logistic regression	Participants < 45 years with low CVH had higher prevalence of depressive symptoms.
Zhao et al., 2024 [34]	Depressive symptoms (PHQ-9 ≥ 10)	CVH; LE8 (low 0–49, moderate 50–79, ideal 80–100	Age, sex, race, poverty income ratio, health insurance, education, marital status, alcohol use	Logistic regression; RCS	Among participants < 40 years, higher LE8 scores were associated with lower odds of depressive symptoms

CES-D-20, 20-item Center for Epidemiologic Studies Depression scale; CES-D-10, 10-item Center for Epidemiological Studies of Depression Scale; CVD, cardiovascular disease; CVH, cardiovascular health; GAD-7, 7-item Generalized Anxiety Disorder; GLMs, generalized linear models; LE8, life’s Essential 8; LS7, Life’s Simple 7; PHQ-9, 9-item Patient Health Questionnaire; RCS, restricted cubic splines; ROC, receiver operating characteristic; STAI-10; 10-item State-Trait Anxiety Inventory;

### Measures of outcomes and exposures

Depressive symptoms were measured using validated tools, including the PHQ-9, 10- and 20-item Center for Epidemiologic Studies Depression scale (CES-D-10, CES-D-20), and self-reported history of diagnosis by a healthcare professional. Anxiety symptoms were assessed using the GAD-7 and a single item from the Centers for Disease Control and Prevention’s Health-Related Quality of Life (HRQoL) Healthy Days Measures. CVH was assessed using the AHA’s LS7, LE8, and modified LS7 metrics. Two studies used the modified LS7: one excluded smoking [35], and the other defined ideal CVH as having 0–1 cardiovascular risk factors [17]. LS7 components are scored as poor (0), intermediate (1), or ideal (2), with the total scores ranging from 0 to 14 and categorised as low (0–7), moderate (8–10), and ideal (11–14) CVH (Table S5). LE8 scores each component from 0 to 100, with the overall score calculated as the average of all eight components and categorised as low (< 50), moderate (50–79), or ideal (≥ 80) [13]. In both metrics, higher scores reflect ideal CVH, although cut-off points may vary across studies. These differences highlight variability in the measurement of both CMDs and CVH across studies.

### Data analysis methods used in included studies

All 11 studies used regression analysis to examine the relationship between CVH and CMDs. Nine applied logistic regression; one study [15] used both logistic and linear regression. Li and Dai [29] used restricted cubic splines (RCS) to assess non-linear relationships, as well as receiver operating characteristics (ROC) curves and DeLong tests to determine predictive ability. Xu et al. [14] and Zhao [34] also applied RCS. Rosal et al. [31] used dominance analysis to identify key predictors of CVH. One study by Zhang et al. [33] used multinomial logistic regression. The cohort study by Carroll et al. [35] combined latent class analysis (LCA) with generalised linear models (GLMs) to identify depressive symptom trajectories and assess their association with CVH at year 20. This study was rated as high quality, receiving seven stars on the NOS [26]. Among cross-sectional studies, eight received five stars, one received six, and one received four, indicating moderate quality based on the modified NOS [27]. Points were commonly lost due to limited information on the characteristics of non-respondents and outcome assessment (Table S6).

### Association between cardiovascular health and common mental disorders

**Fig. 2:**
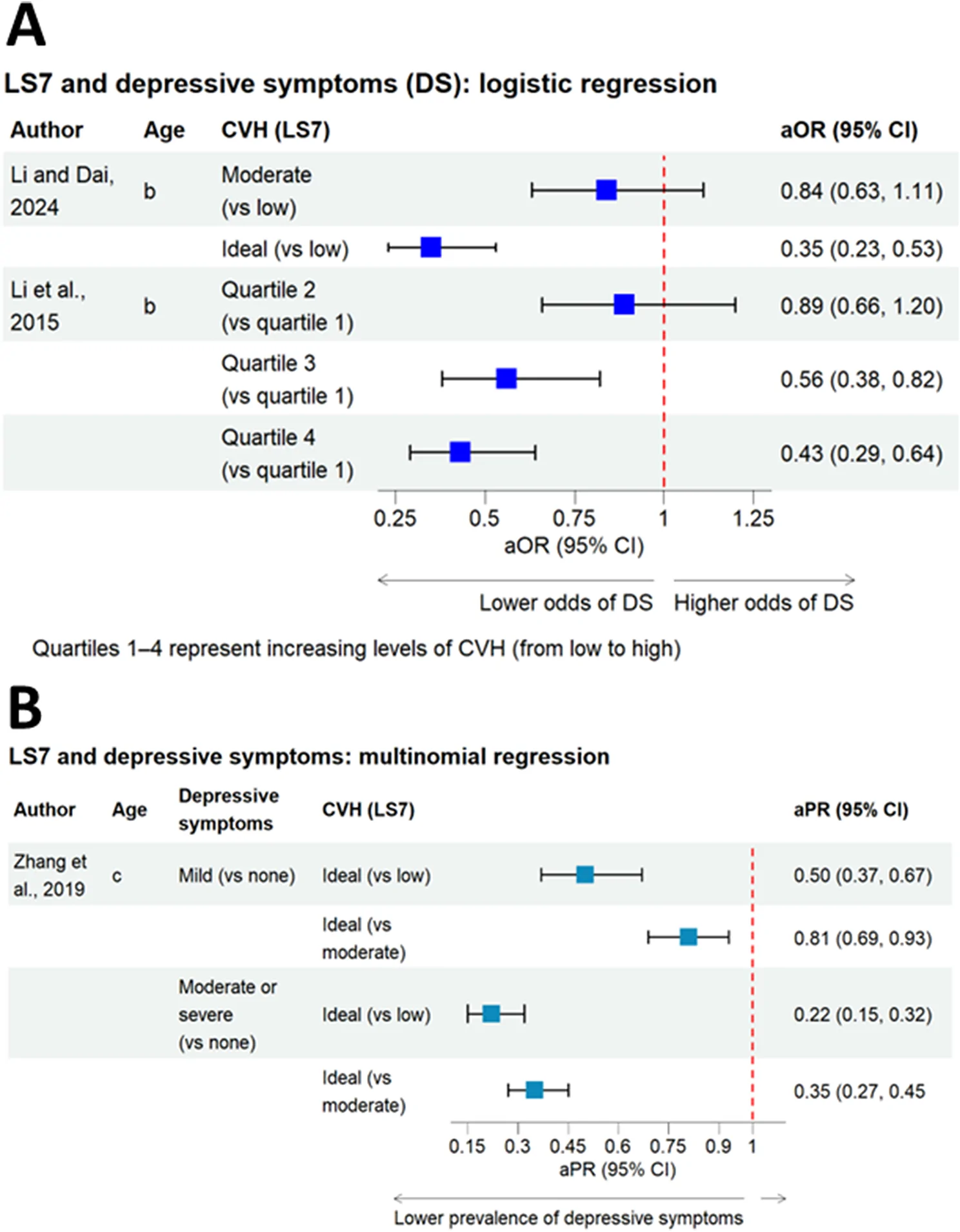
Forest plot of the association between Life’s Simple 7 and depressive symptoms. aPR, adjusted prevalence ratio; aOR, adjusted odds ratio; CI, confidence interval; LS7, Life’s Simple 7; CVH, cardiovascular health. Age groups: b, 20–39 years; c, 20–44 years

**Fig. 3:**
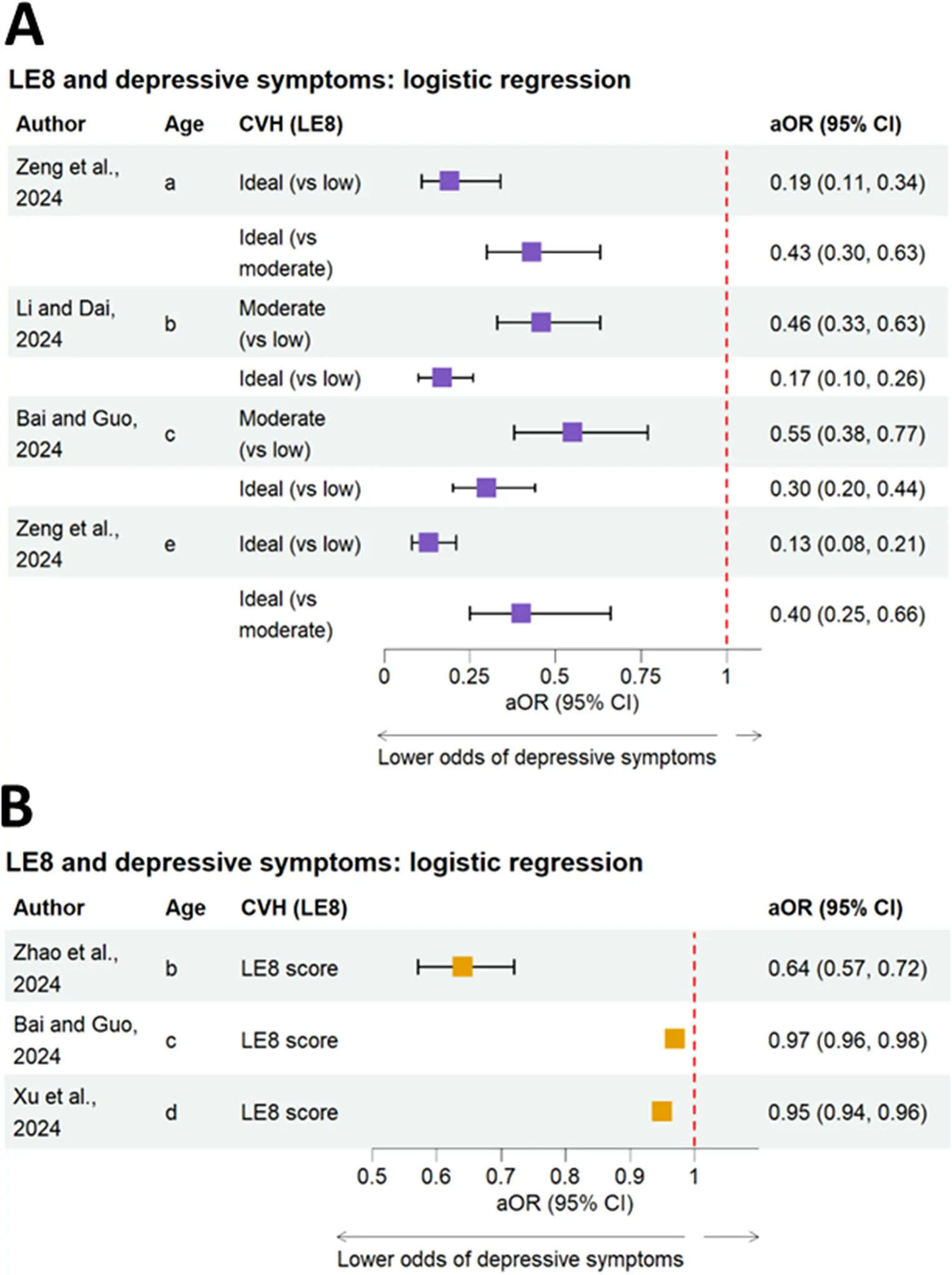
Forest plot of the association between Life’s Essential 8 and depressive symptoms. aOR, adjusted odds ratio; CI, confidence interval; LE8, Life’s Essential 8; CVH, cardiovascular health. Age groups: a, 20–34 years; b, 20–39 years; c, 20–44 years; d, 20–47 years; e, 35–49 years

Seven of the eleven studies examined cross-sectional associations between CVH and CMDs. All studies consistently showed that lower CVH was associated with a higher burden of CMDs. Studies adjusted for sociodemographic, socioeconomic, behavioural, and clinical factors (Table 1b). Most adjusted for age, sex or gender, race or ethnicity, and education. Several also adjusted for marital status, income, poverty indicators, and health insurance, while lifestyle factors (e.g., alcohol and energy intake) and clinical comorbidities were less consistently included. Effect sizes and 95% confidence intervals (CIs) are presented in Fig. 2 and 3.

#### Depressive symptoms outcomes

Figure 2 and 3 demonstrates a consistent association across studies using both LS7 and LE8 metrics, where higher CVH is associated with lower odds and prevalence of depressive symptoms. This inverse association was observed across all reported age groups. For clarity of interpretation, some adjusted odds ratios (aORs) and adjusted prevalence ratios (aPRs) were re-expressed, where appropriate, to ensure a comparable direction.

### LS7 defined CVH

Figure 2A and B present results using the LS7 metric. Among individuals aged 20–39 years, Li and Dai [29] reported reduced odds of depressive symptoms in those with moderate (aOR = 0.46) and ideal (aOR = 0.17) CVH. Similarly, Li et al. [30] found that individuals in quartiles 3 (LS7 score = 11) and 4 (LS7 score = 12–14) had lower odds than those in quartile 1 (LS7 score = 0–8), with aORs of 0.56 and 0.43, respectively. Zhang et al. [33] reported that young adults aged 20–44 years with ideal CVH had a lower prevalence of depressive symptoms compared to those with low or moderate CVH. Specifically, ideal CVH was associated with a lower prevalence of mild depressive symptoms (aPR = 0.50 and 0.81), and a substantially lower prevalence of moderate or severe symptoms (aPR = 0.22 and 0.35, respectively; Fig. 2B).

### LE8 defined CVH

Figure 3A and B present results using LE8 metrics. Bai and Guo [28] found that adults aged 20–44 years with moderate (aOR = 0.55) and ideal CVH (aOR = 0.30) had lower odds of depressive symptoms compared with low CVH. Similar findings were reported by Li and Dai [29] among individuals aged 20–39 years. Zeng et al. [32] observed lower odds of depressive symptoms among individuals aged 20–34 years and 35–49 years with ideal CVH compared to those with low or moderate CVH. Stronger associations were observed among individuals aged 35–49 years compared to those aged 20–34 years. Additionally, Bai and Guo [28], Xu et al. [14], and Zhao et al. [34] reported that each 10-point increase in LE8 score was associated with reduced odds of depressive symptoms across age groups (Fig. 3B).

#### Anxiety symptoms outcomes

Only one study examined anxiety symptoms. Xu et al. [14] reported that each 10-point increase in LE8 score was associated with lower odds of anxiety symptoms among individuals aged 20–47 years (aOR = 0.98; 95% CI: 0.97–0.98).

### Association between common mental disorders and cardiovascular health

**Fig. 4:**
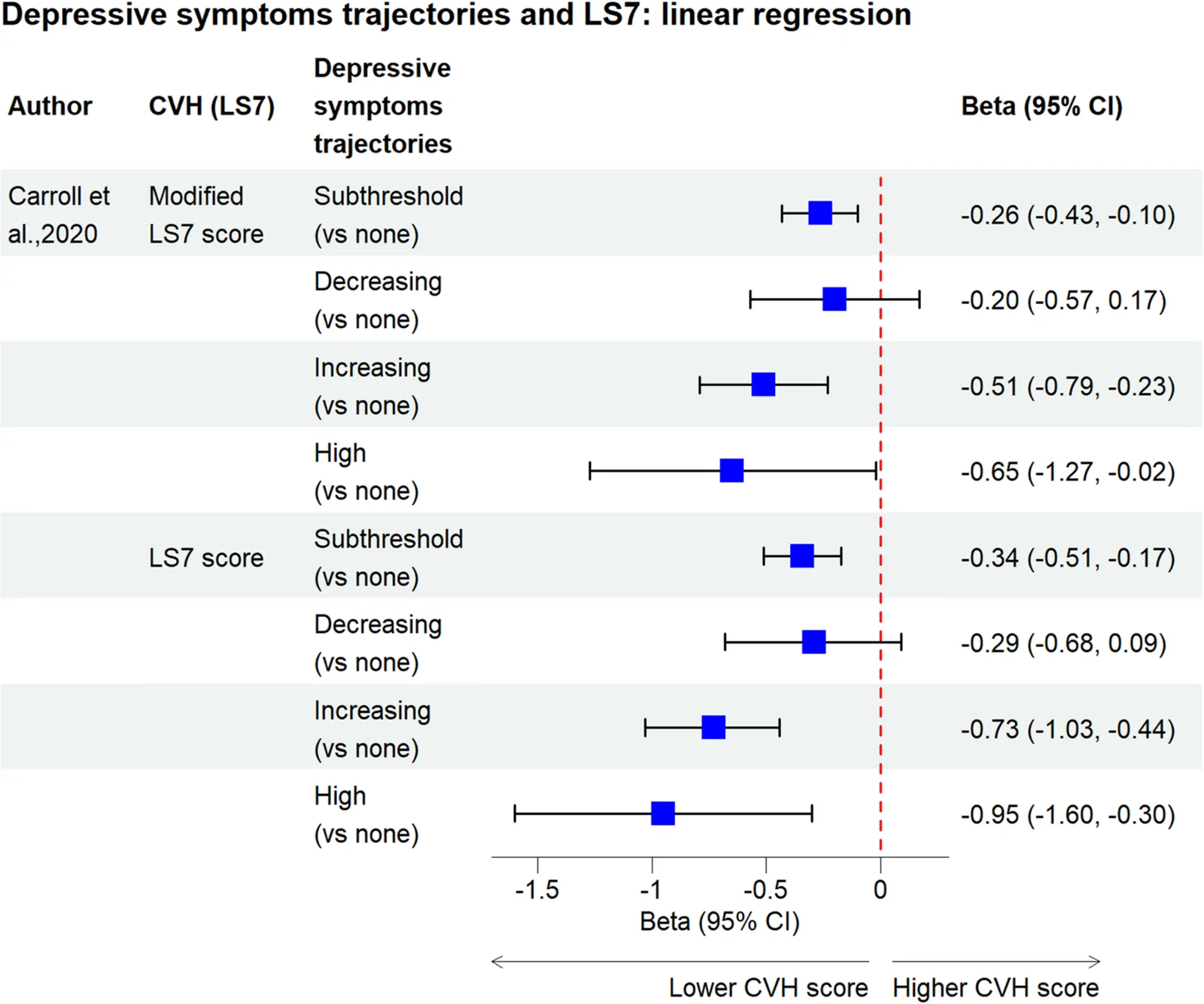
Forest plot of the association between depressive symptoms trajectories and cardiovascular health. CI, confidence interval; CVH, cardiovascular health; LS7, Life’s Simple 7. Participants were aged 18–30 years at baseline and 38–50 years at the 20-year-follow-up. Depressive symptom trajectories over 20 years: consistently low scores (no symptoms), persistent moderate scores below the clinical threshold (subthreshold), initially high scores that declined (decreasing), initially moderate scores that worsened (increasing), and persistently high scores (high symptoms)

**Fig. 5:**
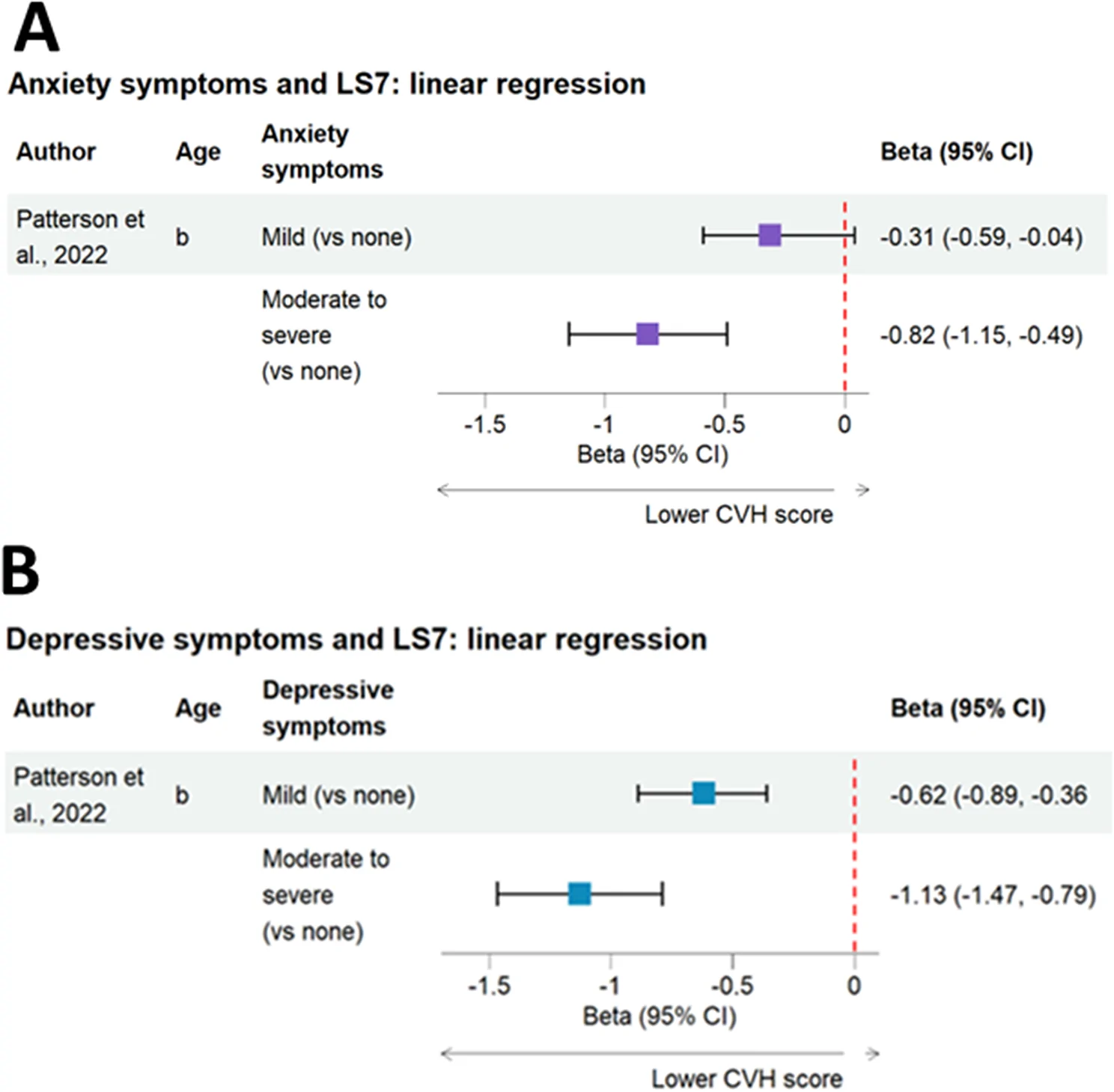
Forest plot of the association of anxiety and depressive symptoms with cardiovascular health. CI, confidence interval; CVH, cardiovascular health; LS7, Life’s Simple 7. Age group: b, 18–34 years

**Fig. 6:**
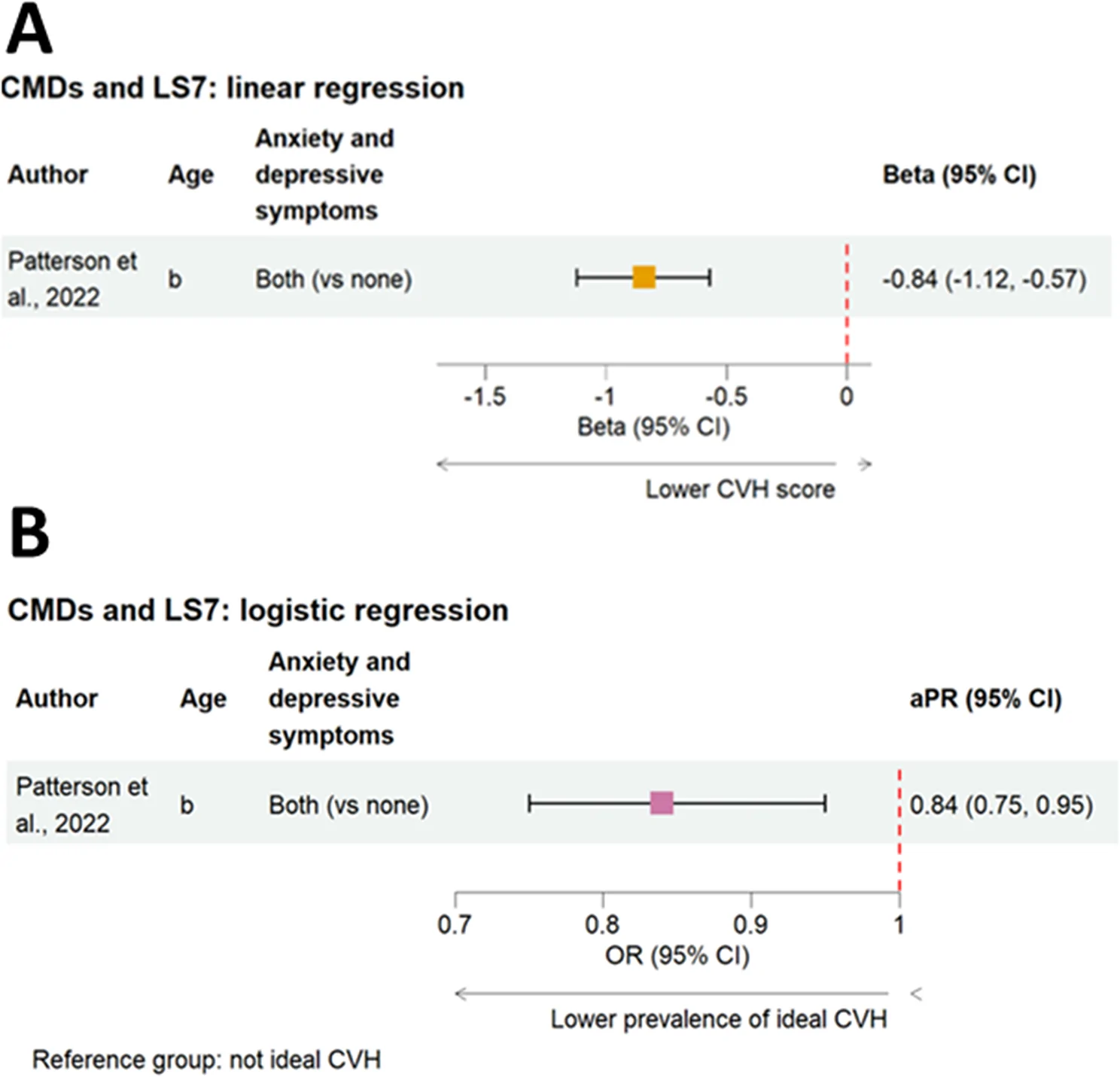
Forest plot of the association of combined anxiety and depressive symptoms with cardiovascular health. aPR, adjusted prevalence ratio; CI, confidence interval; CVH, cardiovascular health; LS7, Life’s Simple 7. Age group: b, 18–34 years

**Fig. 7:**
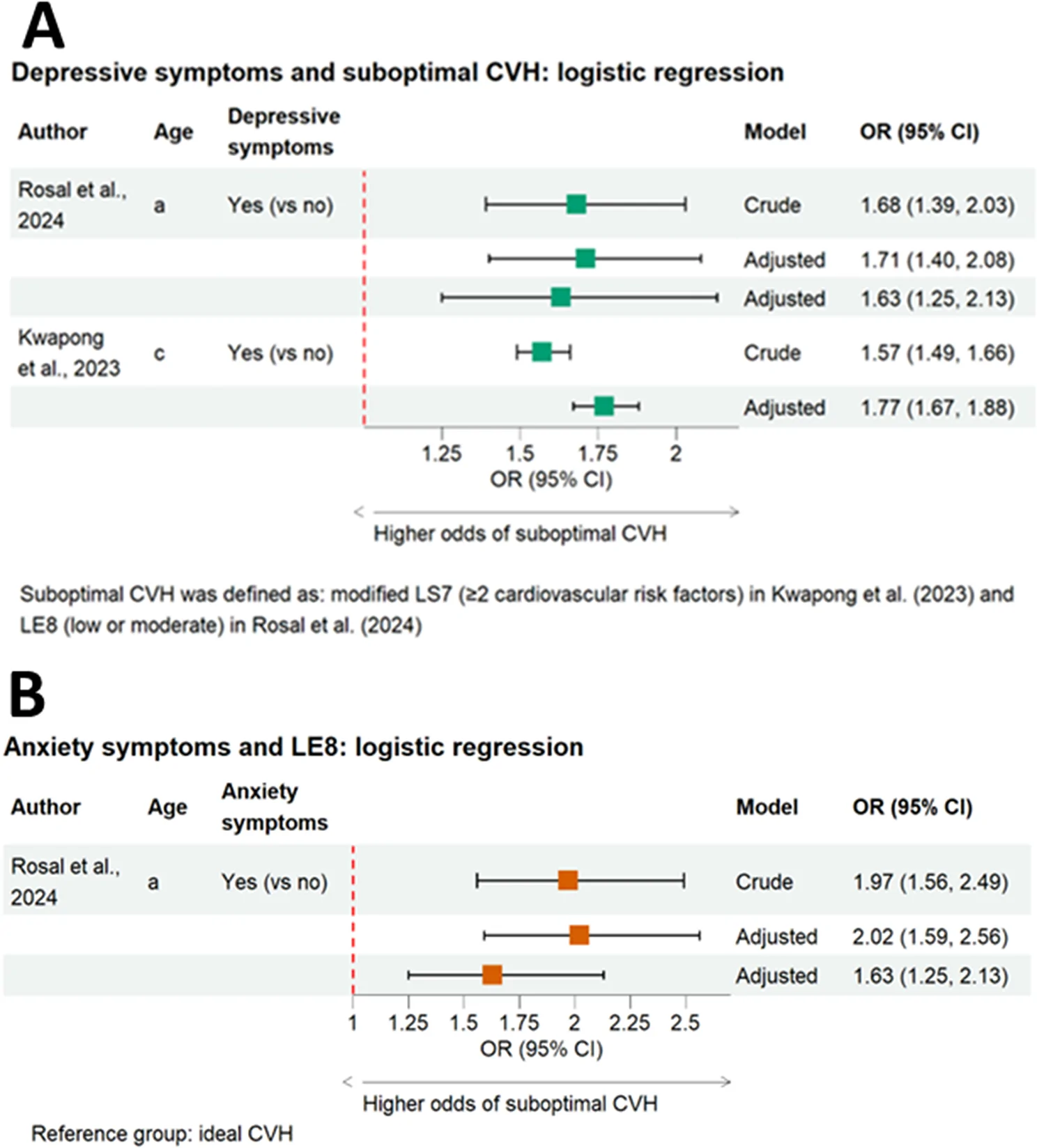
Forest plot of the association of anxiety and depressive symptoms with suboptimal cardiovascular health. OR, odds ratio; CI, confidence interval; CVH, cardiovascular health; LE8, Life’s Essential 8; LS7, Life’s Simple 7. Age groups: a, 18–29 years; c, 18–49 years

Four studies examined associations between CMDs and CVH and consistently showed that CMDs were associated with lower CVH scores, higher odds of suboptimal CVH, or lower likelihood of ideal CVH (Figs. 4, 5, 6 and 7). All adjusted for key sociodemographic variables, including age, sex or gender, race or ethnicity, and education. Two studies additionally adjusted for marital status. Socioeconomic indicators were variably included, such as income, employment status, social status, and health insurance coverage. One study further adjusted for psychological factors (Table 1b).

#### Longitudinal analysis

Figure 4 presents findings from a cohort study by Carroll et al. [35], which assessed the association between depressive symptom trajectories over 20 years and CVH at year 20. Individuals with subthreshold, increasing, and high depressive symptom trajectories had lower CVH scores on the modified LS7 (least squares mean (Ismean) = 6.96, 6.71, and 6.58, respectively) compared to those with no symptoms (lsmean = 7.23), and similar results were observed using the full LS7 metric.

#### Cross-sectional analysis

Figure 5, 6, and 7 summarises cross-sectional associations from three studies.

### Continuous cardiovascular health outcomes

Patterson et al. [15] reported that individuals aged 18–34 years with mild (β = -0.31) and moderate to severe (β = -0.82) anxiety symptoms had lower CVH scores compared to those with no symptoms (Fig. 5A). Similarly, depressive symptoms were associated with lower CVH scores, with β coefficients of -0.62 for mild symptoms and − 1.13 for moderate to severe symptoms (Fig. 5B). Individuals with both anxiety and depressive symptoms also had lower CVH scores (β = -0.84; Fig. 6A).

### Suboptimal cardiovascular health outcomes

Depressive symptoms were associated with higher odds of suboptimal CVH, defined as having ≥ 2 cardiovascular risk factors or low-to-moderate CVH based on LS7 or LE8 metrics. Kwapong et al. [17] reported increased odds among individuals aged 18–49 years (OR = 1.57; aOR = 1.77), while Rosal et al. [31] observed similar associations among those aged 18–29 years (OR = 1.68; aOR = 1.71; aOR = 1.63; Fig. 7A). Anxiety symptoms were also associated with higher odds of suboptimal CVH (OR = 1.97; aOR = 2.02; aOR 1.63; Fig. 7B). Using dominance analysis, Rosal et al. [31] further identified anxiety symptoms (standardised dominance statistic = 0.30) as the most influential predictor of suboptimal CVH, followed by depressive symptoms (0.45).

### Ideal cardiovascular health outcomes

Patterson et al. [15] reported that individuals aged 18–34 years with both anxiety and depressive symptoms had a lower prevalence of ideal CVH compared with those with no symptoms (aPR = 0.84; Fig. 6B).

## Discussion

### Interpretation of findings

This review included 11 observational studies with 731,650 participants. Mean ages ranged from 22 to 48 years. The magnitude of effect sizes varied across the included studies. Seven cross-sectional studies consistently showed that higher CVH was associated with lower odds or prevalence of depressive symptoms. This inverse association was observed across different age groups, CVH metrics, and modelling approaches. One study similarly reported that higher CVH scores were associated with lower odds of anxiety symptoms. Some evidence also suggested stronger associations among older age groups, although this was limited to a single study. Conversely, four studies, including three cross-sectional and one cohort, found that depressive and anxiety symptoms were linked to lower CVH scores, higher odds of suboptimal CVH, or lower prevalence of ideal CVH. Similar patterns were observed for individuals with both anxiety and depressive symptoms. In one study, dominance analysis suggested that anxiety symptoms were the strongest predictor of suboptimal CVH. Overall, findings suggest a potential bidirectional relationship, though this is inferred from separate analyses rather than being directly established within individual studies.

These findings are consistent with previous literature. Our results align with those of Ogunmoroti et al. [16], who reported a bidirectional association between depressive symptoms and CVH, with low CVH linked to depressive symptoms and vice versa. Similarly, Chaplin et al. [36] found that individual cardiovascular risk factors, including BMI, smoking, and blood pressure, were associated with depression in young adults, supporting our observation that lower CVH is associated with depressive symptoms. Although few reviews examined the association between CVH and CMDs, some studies have quantitatively assessed associations between CVDs and CMDs. Zeng et al. [37], in a meta-analysis, reported high prevalence of depressive (20.8%) and anxiety (23.2%) symptoms among individuals with CVDs such as heart failure and coronary heart disease. Their Mendelian randomisation analysis provided stronger genetic evidence for depression contributing to CVD development (OR = 1.168; 95% CI: 1.073–1.270), while the reverse pathway was weaker and more evident for individual conditions such as hypertension and heart failure [37]. Similarly, Krittanawong et al. [5], in a meta-analysis of over 1.9 million individuals, found depression to be associated with increased risk of CVD outcomes, such as stroke, myocardial infarction, and heart failure (hazard ratio [HR] = 1.16; 95% CI: 1.04–1.30). Batelaan et al. [6] reported that anxiety was linked to higher CVD risk (HR = 1.52; 95% CI: 1.36–1.71). These findings support the direction observed in our review, where CMDs are associated with suboptimal CVH. They also highlight the need for integrated screening, prevention, and management strategies.

### Implications for research

Variation in the magnitude of effect sizes across the included studies likely reflects heterogeneity in CVH definitions (including differences in scoring of LS7 and LE8 metrics), CMD assessment tools, and population characteristics. In line with this, all included studies primarily used regression analysis, mostly logistic, with limited use of LCA (to identify symptom trajectories), RCS (to model non-linear associations), and dominance analysis (to quantify variable importance). Although regression remains central to epidemiologic research, reliance on a single family of methods limits exploration of latent subgroups, indirect pathways, and complex interactions inherent in the CVH-CMD relationship. Regression models are powerful for estimating direct associations between observed variables [38]. However, they rely on correct model specification, including assumptions about how variables relate to one another, which limits the ability to capture latent constructs or complex bidirectional processes.

Building on these observations, Rod et al. [39] advocate for methodological pluralism in epidemiology to better address complex health problems. In line with this, approaches including structural equation modelling (SEM), LCA, machine learning, and causal inference offer opportunities for flexible modelling of complex, non-linear, and potential bidirectional relationships [39]. SEM, particularly when applied to longitudinal data, can model direct and indirect effects, latent constructs, and complex pathways, providing a framework for exploring the multifactorial CVH-CMD link [40]. LCA can identify unobserved subgroups or profiles, enabling class-specific associations and trajectory analyses that reveal heterogeneity [39]. Machine-learning approaches, including neural networks, can detect hidden patterns, interactions, and non-linear associations in large datasets, although they also require careful validation to avoid overfitting [39]. Causal inference frameworks can better address issues like time-varying confounding and clarify potential causal pathways, which are difficult to handle using traditional regression approaches [39]. These approaches do not replace regression but can enhance understanding of the dynamic relationships between CVH and CMDs. Future research should embrace methodological pluralism to better reflect the multifaceted nature of these relationships and strengthen both explanatory power and intervention design.

### Implications for practice

Low CVH and CMDs are each associated with reduced quality of life and increased risk of long-term morbidity and mortality [2, 13, 41, 42]. Consistent evidence shows that both conditions increase the risk of chronic diseases (such as CVDs, cancer, kidney disease) and premature mortality [13, 41, 42]. The potential bidirectional associations identified in this review suggest that their co-occurrence may compound these risks. These insights highlight the need for public health interventions that promote healthy behaviours and mental wellbeing, supported not only through individual-level programmes but also through broader structural and environmental policies. Such measures include creating safe and accessible spaces for physical activity and enabling healthier food environments through regulation, pricing, and public awareness. For adolescents and young adults, school-based programme, including physical activity and stress management interventions, strengthened school health services, and improved school food programmes, can support both CVH and mental wellbeing [9, 43]. For adults, workplace wellness initiatives, caregiver support, peer support programmes, and community health campaigns are recommended [44, 45]. In line with the WHO Global Action Plan for NCDs 2013–2030 [46], such strategies could reduce exposure to modifiable risk factors and mitigate the long-term burden of both conditions.

Beyond their individual health impacts, the combined CVH–CMD risk profile can contribute to more complex clinical needs over time. In primary care, this may manifest as greater difficulty with treatment adherence and lifestyle modification [11]. These challenges highlight the importance of integrated prevention and care strategies that address both mental wellbeing and cardiovascular risk. One effective example is the collaborative care model, which has demonstrated improvements in both depressive symptoms and cardiometabolic outcomes among adults with depressive disorder and poorly controlled diabetes [47]. Even when full integration is not feasible, providing individual-level behavioural support in primary care, such as structured physical activity counselling or dietary guidance, remains a practical strategy to improve outcomes across both conditions [48, 49].

### Limitations

This review examined associations between CVH and CMDs among adults aged 15–50 years, offering insight into a life stage where risk trajectories for both conditions often begin. By exploring associations in both directions, it offers a broader understanding of the CVH-CMD relationship. However, most included studies were cross-sectional, limiting causal and temporal inference. Only one longitudinal study, which assessed CMD trajectories from age 18–30 and their association with CVH at year 20 (ages 38–50), offered limited temporal insight. Heterogeneity in study designs and measurement tools precluded meta-analysis, making effect size estimation difficult. Although effect estimates were re‑expressed to ensure consistent directionality, differences in study design and measurement approaches may still limit direct comparability across studies. In addition, the evidence base for associations from CMDs to CVH was limited. Only a small number of studies contributed to each outcome category (continuous CVH scores, suboptimal CVH, and ideal CVH), which restricts the strength and interpretability of subgroup findings. In addition, the possibility of reporting bias cannot be excluded. Studies reporting statistically significant or stronger associations may be more likely to be published, while those with null or weaker findings may be underrepresented, which could influence the overall interpretation of the evidence. CMDs were assessed using diverse instruments, including validated scales such as PHQ-9, CES-D-10, and GAD-7, introducing variability in measurement precision and construct validity. This limited comparability, replicability, and generalisability across contexts. Geographic representation was narrow, with only one study from a low- and middle-income country, restricting applicability to low-resource settings. Furthermore, few studies focused specifically on the 15–50-year age range; most included participants aged 18 years and older, meaning that younger individuals within this age range were generally underrepresented. Most studies used population-based samples, which represent the general population rather than clinic-based cohorts. This limits insight into diagnosed or treatment-seeking populations. In addition, assessment using the NOS indicated that several studies had limitations across key domains. In the selection domain, many studies did not report the characteristics of non-respondents, raising the possibility of selection bias. In the outcome assessment domain, reliance on self-reported measures of CMDs and CVH may have introduced measurement bias. These factors should be considered when interpreting the consistency and strength of the observed associations. Although associations were observed in both directions, no study explicitly assessed bidirectionality. As such, the bidirectional relationship between CVH and CMDs is inferred from separate analyses conducted in different studies rather than directly tested with the same population. This limits the ability to draw firm conclusions about the bidirectional relationship.

The review has several methodological limitations. Full-text screening, data extraction, and quality appraisal were conducted by a single reviewer, with oversight from co-authors, which may still increase the risk of selection or extraction bias. Although this oversight and quality checking may have helped reduce potential errors, the lack of independent verification may have affected the consistency, completeness, and accuracy of study inclusion and extracted data, potentially influencing the interpretation and synthesis of findings. The exclusion of grey literature may have resulted in relevant studies being missed. In addition, reference list screening was not performed, which may have increased the risk of missing relevant studies. Furthermore, heterogeneity across study designs and outcome measures precluded meta-analysis. These limitations should be considered when interpreting the findings.

## Conclusions

This review suggests a potential bidirectional relationship between CVH and CMDs among adults, with higher CVH associated with a lower burden of CMDs, and CMDs associated with lower CVH. These findings underscore the importance of integrated approaches that address both cardiovascular and mental health. At the individual level, strategies such as promoting physical activity, healthy diet, and stress management can benefit both CVH and CMDs. At the community level, school-based programmes for adolescents, workplace wellness initiatives, and community health campaigns can foster healthy behaviours and psychological wellbeing. At the policy level, creating supportive environments, through safe spaces for physical activity and improved access to healthy foods, can enable healthier choices and reduce long-term risk exposure. In primary care, collaborative care models offer practical opportunities for integrated prevention and management. Policy support for integrated care models could further enhance implementation. Given the predominance of cross-sectional designs and limited methodological diversity, future research should prioritise longitudinal studies and embrace methodological pluralism. Methods such as causal inference, SEMs, LCA, and machine learning could clarify directionality and complexity.

## Supplementary Information

Below is the link to the electronic supplementary material.

Supplementary Material 1

## Data Availability

No datasets were generated or analysed during the current study.
