# Supplementary material for: The relationship between cardiovascular health and common mental disorders among adults aged ≤ 50 years: a systematic review of observational studies
Source: BMC Psychiatry. 2026 Jun 16;26:663. doi: 10.1186/s12888-026-08281-w (PMC13505032; doi:10.1186/s12888-026-08281-w)
Supplement: Supplementary file 1 [file 12888_2026_8281_MOESM1_ESM.docx]

**Table S1: MEDLINE (via PubMed) database search strategy**

|  | **MEDLINE (via PubMed)** | **Results**  **(04/11/2024)** |
| --- | --- | --- |
| 1 | "Mental health" [MeSH Terms] OR "mental disorders" [MeSH Terms] OR depress*[MeSH Terms] OR anxiety [MeSH Terms] OR "mood disorders" [MeSH Terms] | 1,710,327 |
| 2 | "Mental health"[Title/Abstract] OR "mental disorder"[Title/Abstract] OR "mental illness*"[Title/Abstract] OR "psychiatric disorder"[Title/Abstract] OR "mental wellbeing"[Title/Abstract] OR "mental well-being"[Title/Abstract] OR depress*[Title/Abstract] OR anxiety [Title/Abstract] OR anxious [Title/Abstract] OR "mood disorder*"[Title/Abstract] OR "affective disorder*"[Title/Abstract] | 988,758 |
| 3 | #1 AND #2 | 2,186,104 |
| 4 | "Heart Disease Risk Factors" [MeSH Terms] | 7,887 |
| 5 | "Cardiovascular health"[Title/Abstract] OR "Cardiovascular health metrics"[Title/Abstract] OR "life’s simple 7"[Title/Abstract] OR "life’s simple seven"[Title/Abstract] OR "life’s essential 8"[Title/Abstract] OR "Cardiovascular risk score*"[Title/Abstract] OR "cardiovascular risk index"[Title/Abstract] OR "Composite cardiovascular risk"[Title/Abstract] OR "combined cardiovascular risk"[Title/Abstract] OR "Combined cardiovascular risk factors"[Title/Abstract] | 16,329 |
| 6 | #4 AND #5 | 23,676 |
| 7 | "Young adult" [MeSH Terms] OR adolescent [MeSH Terms] OR adult [MeSH Terms] | 8,941,567 |
| 8 | youth*[Title/Abstract] OR "young people"[Title/Abstract] OR adolescent*[Title/Abstract] OR adolescence [Title/Abstract] OR teen*[Title/Abstract] OR "young person"[Title/Abstract] OR adult*[Title/Abstract] | 2,092,520 |
| 9 | #7 AND #8 | 9,793,091 |
| 10 | #3 AND #6 AND #9 | 1,290 |

**Table S2: CINAHL (EBSCO) database search strategy**

|  | **Theme** | **CINAHL** | **Results**  **(04/11/2024)** |
| --- | --- | --- | --- |
| 1 | **CMDs**  Any Field | “Mental health” OR “mental disorder” OR “mental illness*” OR “psychiatric disorder” OR “mental wellbeing” OR “mental well-being” OR depress* OR anxiety OR anxious OR “mood disorder*” OR “affective disorder*” | 490,274 |
| 2 | **Cardiovascular Health**  Any Field | “Cardiovascular health” OR “Cardiovascular health metrics” OR “life’s simple 7” OR “life’s simple seven” OR “life’s essential 8” OR “Cardiovascular risk score*” OR "cardiovascular risk index" OR "Composite cardiovascular risk" OR "combined cardiovascular risk" OR “Combined cardiovascular risk factors” | 6,373 |
| 3 | **Young adults**  Any Field | youth* OR “young people” OR adolescent* OR adolescence OR teen* OR “young person” OR adult* | 1,933,068 |
| 4 |  | #1 AND #2 AND #3 | 230 |

**Table S3: Web of Science database search strategy**

|  | **Theme** | **Web of Science** | **Results**  **04/11/2024** |
| --- | --- | --- | --- |
| 1 | **CMDs**  Topic | **TS**= (“Mental health” OR “mental disorder” OR “mental illness*” OR “psychiatric disorder” OR “mental wellbeing” OR “mental well-being” OR depress* OR anxiety OR anxious OR “mood disorder*” OR “affective disorder*”) | 1,395,856 |
| 2 | **Cardiovascular Health**  Topic | **TS**= (“Cardiovascular health” OR “Cardiovascular health metrics” OR “life’s simple 7” OR “life’s simple seven” OR “life’s essential 8” OR “Cardiovascular risk score*” OR "cardiovascular risk index" OR "Composite cardiovascular risk" OR "combined cardiovascular risk" OR “Combined cardiovascular risk factors” ) | 19,737 |
| 3 | **Young adults**  Topic | **TS**= (youth* OR “young people” OR adolescent* OR adolescence OR teen* OR “young person” OR adult*) | 2,885,568 |
| 4 |  | #1 AND #2 AND #3 | 585 |

**Table S4: APA PsycINFO database search strategy**

|  | **Theme** | **PsycINFO** | **Results**  **04/11/2024** |
| --- | --- | --- | --- |
| 1 | **Mental health**  Either Title or Abstract | **Title**: “Mental health” OR “mental disorder” OR “mental illness*” OR “psychiatric disorder” OR “mental wellbeing” OR “mental well-being” OR depress* OR anxiety OR anxious OR “mood disorder*” OR “affective disorder*” *OR* **Abstract**: “Mental health” OR “mental disorder” OR “mental illness*” OR “psychiatric disorder” OR “mental wellbeing” OR “mental well-being” OR depress* OR anxiety OR anxious OR “mood disorder*” OR “affective disorder*” | 755,990 |
| 2 | **Cardiovascular Health/ Cardiovascular diseases**  Either Title or Abstract | **Title:** “Cardiovascular health” OR “Cardiovascular health metrics” OR “life’s simple 7” OR “life’s simple seven” OR “life’s essential 8” OR “Cardiovascular risk score*” OR "cardiovascular risk index" OR "Composite cardiovascular risk" OR "combined cardiovascular risk" OR “Combined cardiovascular risk factors” OR **Abstract:** “Cardiovascular health” OR “Cardiovascular health metrics” OR “life’s simple 7” OR “life’s simple seven” OR “life’s essential 8” OR “Cardiovascular risk score*” OR "cardiovascular risk index" OR "Composite cardiovascular risk" OR "combined cardiovascular risk" OR “Combined cardiovascular risk factors” | 2,041 |
| 3 | **Young adults**  Either Title or Abstract | **Title:** youth* OR “young people” OR adolescent* OR adolescence OR teen* OR “young person” OR adult* OR **Abstract:** youth* OR “young people” OR adolescent* OR adolescence OR teen* OR “young person” OR adult* | 853,184 |
| 7 |  | #1 AND #2 AND #3 | 126 |

**Text S1: Newcastle-Ottawa Scale for cohort and modified version for cross-sectional studies**

**NEWCASTLE - OTTAWA QUALITY ASSESSMENT SCALE COHORT STUDIES**

Note: A study can be awarded a maximum of one star for each numbered item within the Selection and Outcome categories. A maximum of two stars can be given for Comparability

**Selection**

1. Representativeness of the exposed cohort
   a) truly representative of the average _______________ (describe) in the community *****
   b) somewhat representative of the average ______________ in the community *****
   c) selected group of users e.g. nurses, volunteers
   d) no description of the derivation of the cohort
2. Selection of the non-exposed cohort
   a) drawn from the same community as the exposed cohort *****
   b) drawn from a different source
   c) no description of the derivation of the non-exposed cohort
3. Ascertainment of exposure
   a) secure record (e.g. surgical records) *****
   b) structured interview *****
   c) written self-report
   d) no description
4. Demonstration that outcome of interest was not present at start of study
   a) yes *****
   b) no

**Comparability**

1. Comparability of cohorts based on the design or analysis
   a) study controls for _____________ (select the most important factor) *****
   b) study controls for any additional factor ***** (This criterion could be modified to indicate specific control for a second important factor.)

**Outcome**

1. Assessment of outcome
   a) independent blind assessment *****
   b) record linkage *****
   c) self-report
   d) no description
2. Was follow-up long enough for outcomes to occur
   a) yes (select an adequate follow up period for outcome of interest) *****
   b) no
3. Adequacy of follow up of cohorts
   a) complete follow up - all subjects accounted for *****
   b) subjects lost to follow up unlikely to introduce bias - small number lost - > ____ % (select an adequate %) follow up, or description provided of those lost) *****
   c) follow up rate < ____% (select an adequate %) and no description of those lost
   d) no statement

**NEWCASTLE - OTTAWA QUALITY ASSESSMENT SCALE**

**(Modified version for cross-sectional studies)**

**Selection:** (Maximum 3 stars)

1. Representativeness of the sample:
   a) Truly representative of the average in the target population. ***** (all subjects or random sampling)
   b) Somewhat representative of the average in the target population. ***** (non-random sampling)
   c) Selected group of users.
   d) No description of the sampling strategy.
2. Non-respondents:
   a) Comparability between respondents and non-respondents’ characteristics is established, and the response rate is satisfactory. *****
   b) The response rate is unsatisfactory, or the comparability between respondents and non-respondents is unsatisfactory.
   c) No description of the response rate or the characteristics of the responders and the non-responders.
3. Ascertainment of the exposure (risk factor):
   a) Validated measurement tool. *****
   b) Non-validated measurement tool, but the tool is available or described.
   c) No description of the measurement tool.

**Comparability:** (Maximum 2 stars)

1. The subjects in different outcome groups are comparable, based on the study design or analysis. Confounding factors are controlled.
a) The study controls for the most important factor (select one). *****
b) The study control for any additional factor. *****

**Outcome:** (Maximum 2 stars)

1. Assessment of the outcome:
   a) Independent blind assessment. *****
   b) Record linkage. *****
   c) Self-report.
   d) No description.
2. Statistical test:

a) The statistical test used to analyze the data is clearly described and appropriate, and the measurement of the association is presented, including confidence intervals and the probability level (p value). *****
b) The statistical test is not appropriate, not described or incomplete.

**References**

1. Wells G, Shea B, O'Connell D, Peterson J, Welch V, Losos M, et al. The Newcastle-Ottawa Scale (NOS) for assessing the quality of nonrandomised studies in meta-analyses. 2011. [https://www.ohri.ca/programs/clinical_epidemiology/oxford.asp](https://www.ohri.ca/programs/clinical_epidemiology/oxford.asp%20) (accessed August 31, 2025).

2. Patra J, Bhatia M, Suraweera W, Morris SK, Patra C, Gupta PC, et al. Exposure to second-hand smoke and the risk of tuberculosis in children and adults: a systematic review and meta-analysis of 18 observational studies. *PLOS Medicine* 2015; 12(6):1–21. <https://doi.org/10.1371/journal.pmed.1001835>

**Table S5: Definition of life’s essential 7 by the American Heart Association**

| **CVH metrics** | **Points** | **Definition** |
| --- | --- | --- |
| Blood pressure | Poor (0) | SBP ≥140 mmHg or DBP ≥90 mmHg |
|  | Intermediate (1) | SBP ≥120 & < 140 mmHg or DBP ≥80 & <90 mmHg, treated |
|  | Ideal (2) | SBP <120 and DBP <80 mmHg, untreated |
| Blood glucose | Poor (0) | ≥126 mg/dL |
|  | Intermediate (1) | 100–125 mg/dL or treated |
|  | Ideal (2) | <100 mg/dL |
| Cholesterol | Poor (0) | ≥240 mg/dL |
|  | Intermediate (1) | <200 mg/dL on medication or ≥200 & <240 mg/dL |
|  | Ideal (2) | <200 mg/dL, untreated |
| Smoking | Poor (0) | Current smoker |
|  | Intermediate (1) | Quit smoking in the past 12 months |
|  | Ideal (2) | Never smoke or quit smoking more than 12 months ago |
| Body mass index | Poor (0) | 30 kg/m^2^ |
|  | Intermediate (1) | ≥25 & < 30 kg/m^2^ |
|  | Ideal (2) | <25 kg/m^2^ |
| Physical activity | Poor (0) | No moderate or vigorous activity |
|  | Intermediate (1) | 1–149 minutes per week of moderate activity, or 1–74 minutes per week of vigorous activity, or 1–149 minutes per week of moderate and vigorous activity |
|  | Ideal (2) | ≥150 minutes per week of moderate activity, or ≥75 minutes per week of vigorous activity, or ≥150 minutes of moderate and vigorous activity |
| Diet | Poor (0) | 0 or 1 components |
|  | Intermediate (1) | 2 or 3 components |
|  | Ideal (2) | 4 or 5 components |
| Adapted from Lloyd Jones et al^1^ and Naman et al^2^.  Abbreviations: DBP, diastolic blood pressure; SBP, systolic blood pressure; mg/dL, milligram per deciliter; mmHg, millimeters of mercury; kg/m^2^, kilograms per meter squared | | |

**References**

1. Lloyd-Jones DM, Hong Y, Labarthe D, Mozaffarian D, Appel LJ, Van Horn L, et al. Defining and setting national goals for cardiovascular health promotion and disease reduction: the American Heart Association's strategic Impact Goal through 2020 and beyond. *Circulation* 2010; 121(4):586–613. <https://doi.org/10.1161/circulationaha.109.192703>
2. Shetty NS, Gaonkar M, Patel N, Li P, Arora G, Arora P. Association of Life’s Essential 8 and Simple 7 scores with Mortality: Comparison with Pooled Cohort Equation. *JACC: Advances* 2024; 3(6):100945. <https://doi.org/10.1016/j.jacadv.2024.100945>

**Table S6: Quality assessment using Newcastle-Ottawa for cohort and cross-sectional studies (modified)**

| **Cohort studies** | | | | | | | | | | |
| --- | --- | --- | --- | --- | --- | --- | --- | --- | --- | --- |
| **Author, year** | **Selection** | | | | **Comparability** | **Outcome** | | | **Total stars** | **Study quality** |
|  | Representativeness of the exposed cohort | Selection of the non-exposed cohort | Ascertainment of exposure | Demonstration that the outcome of interest was not present at start of study | Comparability of cohorts based on the design or analysis | Assessment of outcome | Was follow-up long enough for outcomes to occur | Adequacy of follow up of cohorts |  |  |
| Carroll et al., 2020 | * | * | * | - | ** | - | * | * | 7 | Moderate |
|  |  |  |  |  |  |  |  |  |  |  |
| **Cross-sectional studies** | | | | | | | | | | |
| **Author (year)** | **Selection** | | | | **Comparability** | **Outcome** | | | **Total stars** | **Study quality** |
|  | Representativeness of the sample | Non-respondents | Ascertainment of the exposure (risk factor) | | Comparability of outcome groups based on design or analysis | Assessment of outcome | Statistical test is appropriate | |  |  |
| Bai & Guo, 2024 | * | - | * | | ** | - | * | | 5 | Moderate |
| Kwapong et al., 2023 | * | - | - | | ** | - | * | | 4 | Moderate |
| Li & Dai, 2024 | * | * | * | | ** | - | * | | 6 | Moderate |
| Li et al., 2015 | * | - | * | | ** | - | * | | 5 | Moderate |
| Patterson et al., 2022 | * | - | * | | ** | - | * | | 5 | Moderate |
| Rosal et al., 2024 | * | - | * | | ** | - | * | | 5 | Moderate |
| Xu et al., 2024 | * | - | * | | ** | - | * | | 5 | Moderate |
| Zeng et al., 2024 | * | - | * | | ** | - | * | | 5 | Moderate |
| Zhang et al., 2019 | * | - | * | | ** | - | * | | 5 | Moderate |
| Zhao et al., 2024 | * | - | * | | ** | - | * | | 5 | Moderate |
